# Supplementary material for: Airspace Diameter Map—A Quantitative Measurement of All Pulmonary Airspaces to Characterize Structural Lung Diseases
Source: Cells. 2023 Sep 28;12(19):2375. doi: 10.3390/cells12192375 (PMC10571657; doi:10.3390/cells12192375)
Supplement: Supplementary file 1 [file cells-12-02375-s001.zip › cells-2513834-supplementary.pdf]

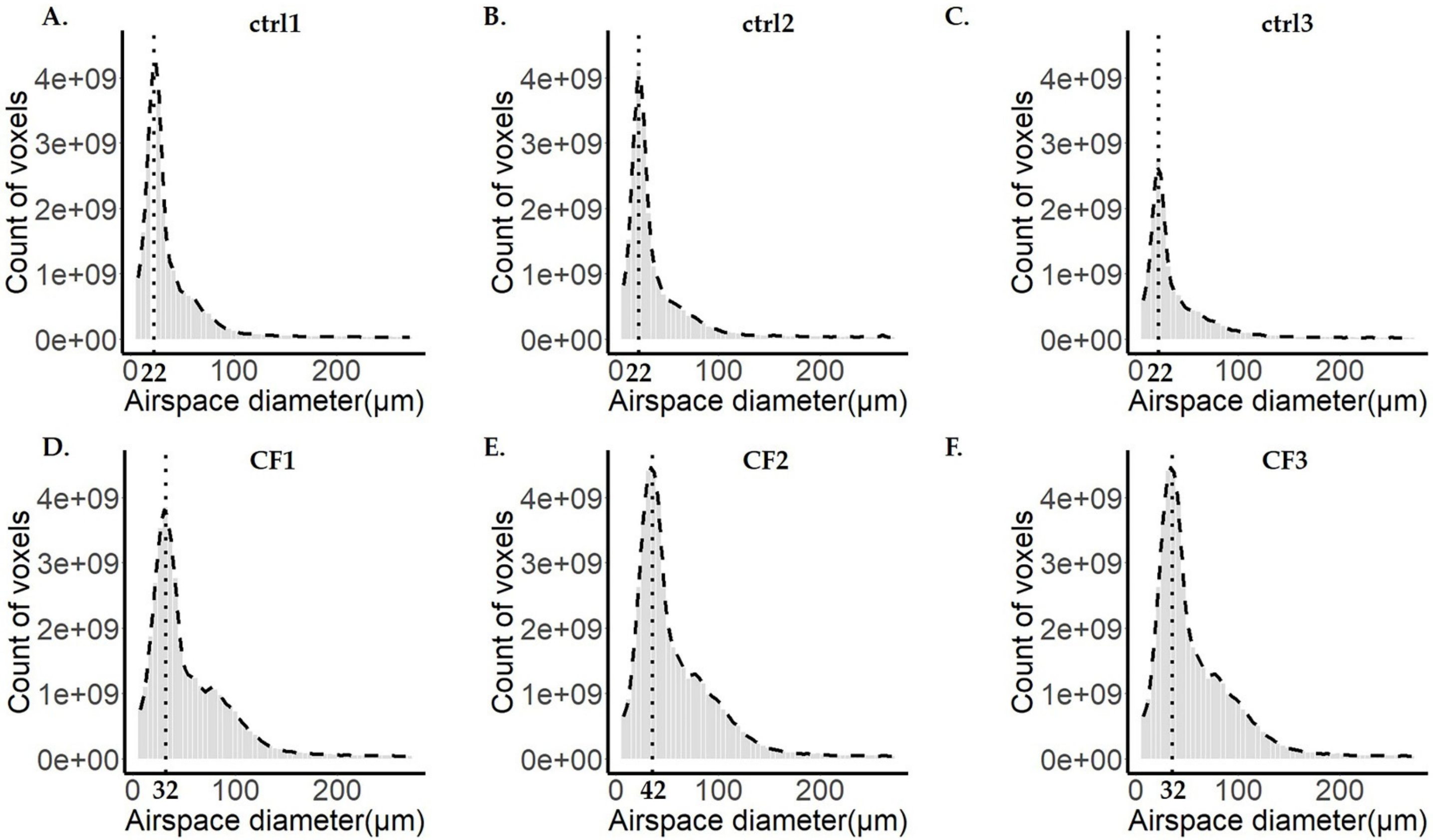

Supplementary Figure S1. The airspace diameter count distribution of ctrl and βENAC-transgenic mice lungs with CF-like disease from pnd36. Fits of histogram distributions of voxel counts are plotted for the control (A-C) and CF lungs (D-F). The corresponding fits are shown with black dashed line. The maximal peak for each fit is labeled with the black dotted line and the value of airspace diameter associated with the maximal peak is labeled in black bold letters. ctrl = control; CF = cystic fibrosis.

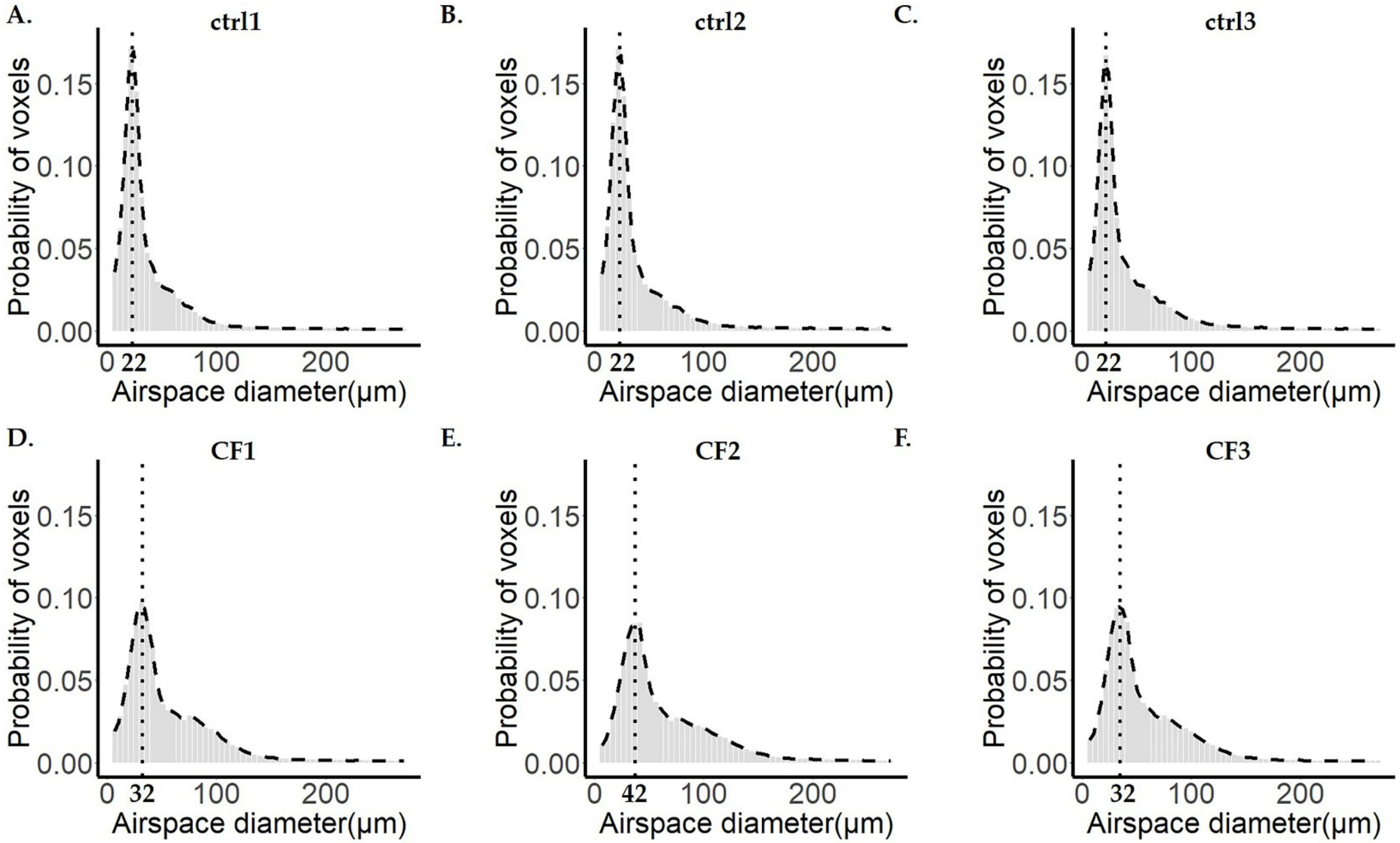

Supplementary Figure S2. The airspace diameter probability distribution of ctrl and βENAC-transgenic mice lungs with CF-like disease from pnd36. Fits of histogram distributions of voxel probability are plotted for the control (A-C) and CF lungs (D-F). The corresponding fits are shown with black dashed line. The maximal peak for each fit is labeled with the black dotted line and the value of airspace diameter associated with the maximal peak is labeled in black bold letters. ctrl = control; CF = cystic fibrosis.

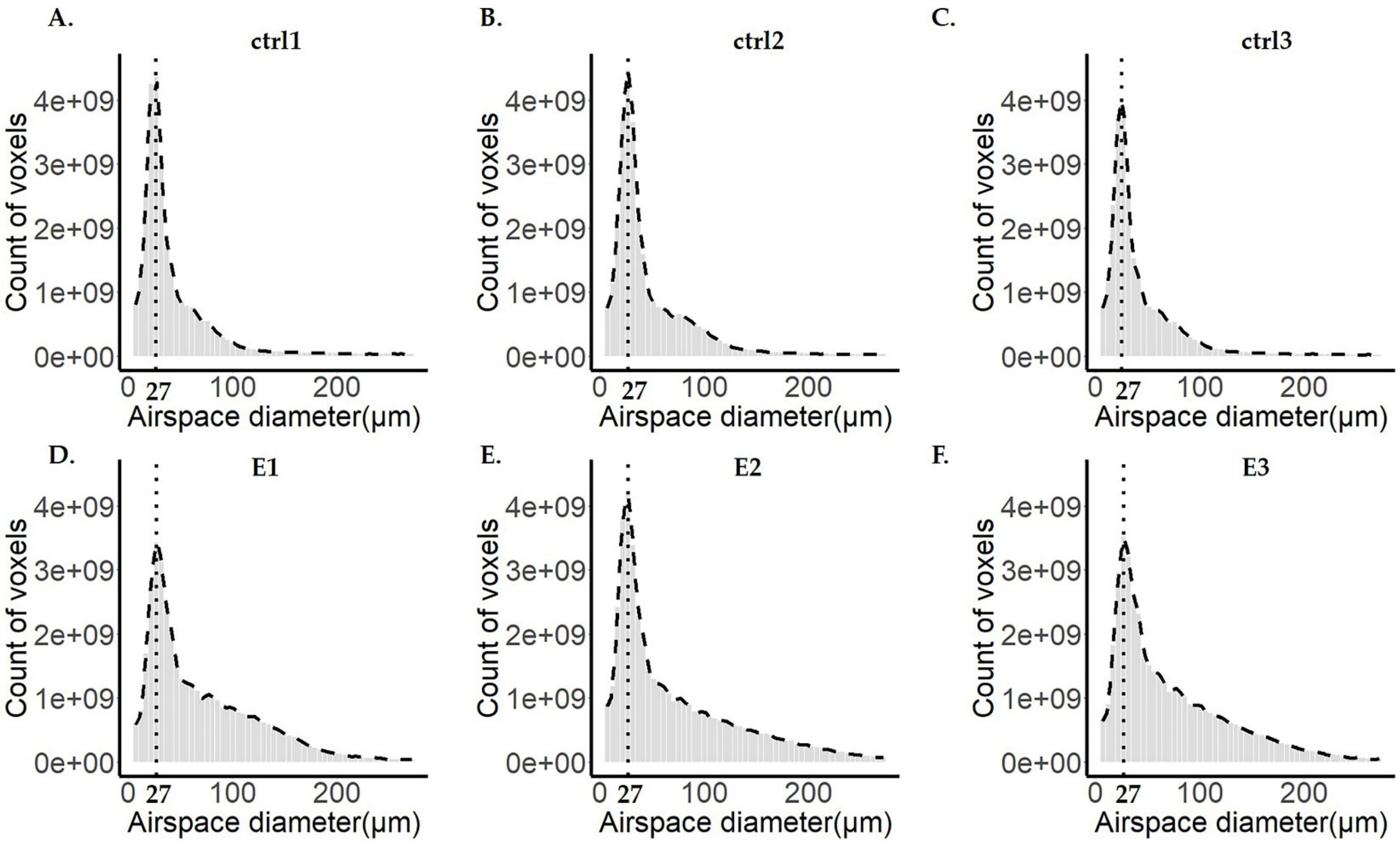

Supplementary Figure S3. The airspace diameter count distribution of ctrl and elastase-instilled mice lungs from pnd100. Fits of histogram distributions of voxel counts are plotted for the control (A-C) and CF lungs (D-F). The corresponding fits are shown with black dashed line. The maximal peak for each fit is labeled with the black dotted line and the value of airspace diameter associated with the maximal peak is labeled in black bold letters. ctrl = control; E = elastase-instilled lung samples.

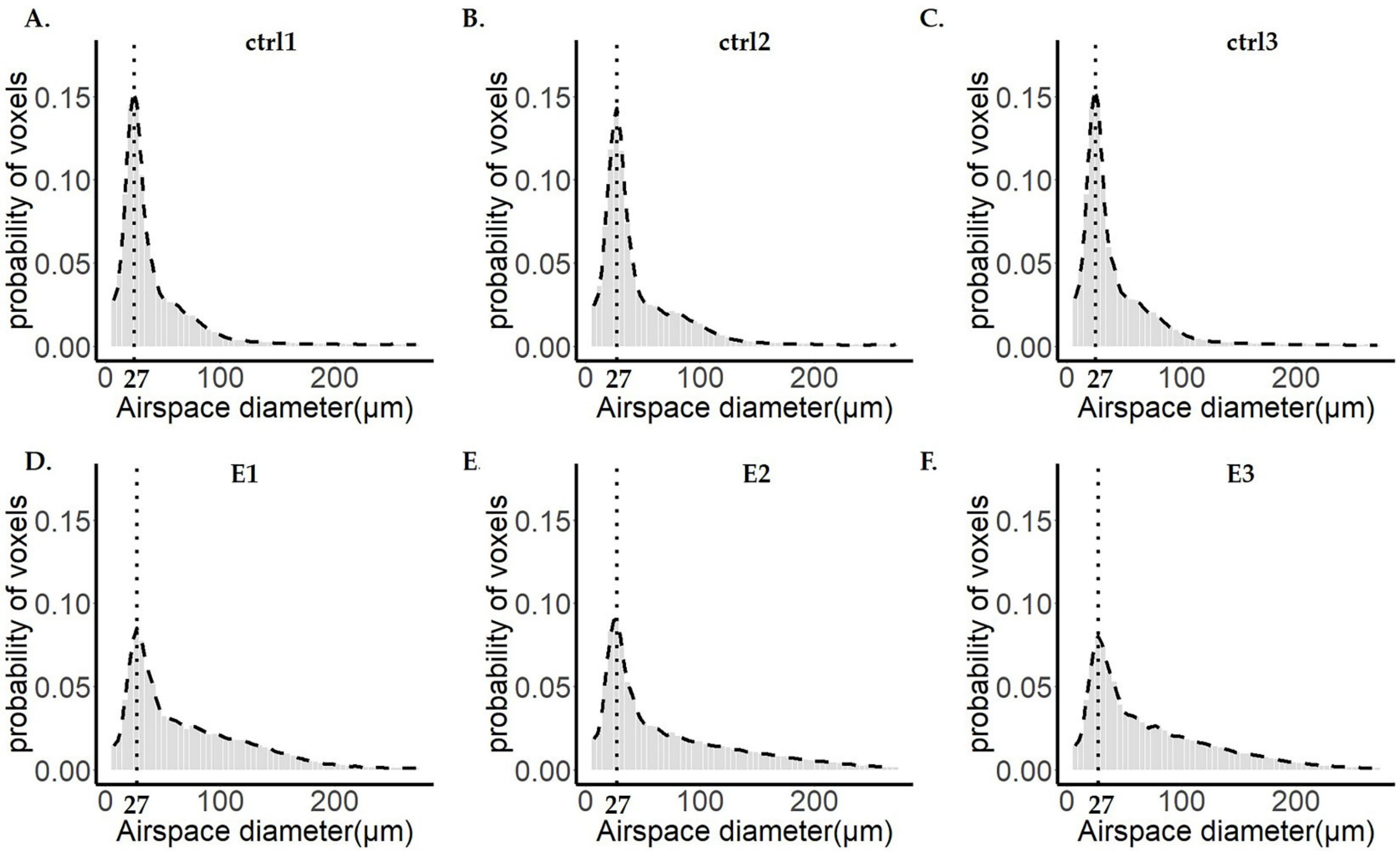

Supplementary Figure S4. The airspace diameter probability distribution of ctrl and elastase-instilled mice lungs from pnd100. Fits of histogram distributions of voxel probability are plotted for the control (A-C) and CF lungs (D-F). The corresponding fits are shown with black dashed line. The maximal peak for each fit is labeled with the black dotted line and the value of airspace diameter associated with the maximal peak is labeled in black bold letters. ctrl = control; E = elastase-instilled lung samples.
